# Patterns and determinants of essential neonatal care utilization among underprivileged ethnic groups in Midwest Nepal: a mixed method study

**DOI:** 10.1186/s12884-019-2465-6

**Published:** 2019-08-27

**Authors:** Keshab Sanjel, Sharad Raj Onta, Archana Amatya, Prem Basel

**Affiliations:** 1 Department of Community Medicine and Public Health, Institute of Medicine, Tribhuvan University, Kathmandu, Nepal; 2 Nepal Public Health Foundation, Kathmandu, Nepal

**Keywords:** Determinants of essential neonatal care, Midwest Nepal, Mixed methods study patterns of essential neonatal care

## Abstract

**Background:**

Globally in 2017 neonatal death accounted for 46% of under-five deaths. Nepal is among the developing countries which has a high number of neonatal deaths. The rates are high among poor socio-economic groups, marginalized, as well as people living in remote areas of Nepal. This paper, thus tries to examine the utilization pattern and maternal, household, and health service factors affecting underprivileged ethnic groups in Midwest Nepal.

**Methods:**

A cross-sectional mixed method study was conducted from September 2017 to April 2018 in Bardiya district. Quantitative data were collected from a household survey of women who gave live births within the last 12 months prior to data collection (*n* = 362). Interviews were also undertaken with 10 purposively selected key informants. Logistic regression model was used to determine the factors associated with essential neonatal care utilization. Thematic analysis was undertaken on the qualitative data.

**Results:**

Overall, neonatal care utilization was 58.6% (53.3–63.7%), with big variations seen in the coverage of selected neonatal care components. Factors such as birth order (2.059, 1.13–3.75), ethnicity (2.28, 1.33–3.91), religion (2.37, 1.03–5.46), perceived quality of maternal and neonatal services (2.66, 1.61–4.39) and awareness on immediate essential newborn cares (2.22, 1.28–3.87) were identified as the determining factors of neonatal care utilization.

**Conclusions:**

The coverage of birth preparedness and complication readiness, adequate breastfeeding, and postnatal care attendance were very low as compared to the national target for each component. The determinants of essential neonatal care existed at maternal, household as well as health facility level and included ethnicity, religion, perceived quality of maternal and neonatal services, birth order and awareness on immediate essential newborn care. Appropriate birth spacing, improving the quality of maternal and neonatal services at health facilities and raising mother’s level of awareness about neonatal care practices are recommended.

**Electronic supplementary material:**

The online version of this article (10.1186/s12884-019-2465-6) contains supplementary material, which is available to authorized users.

## Background

The neonatal period- the first 28 days of life, is the most critical time for a child’s growth and survival. Globally, 46% of under-five deaths fall in the neonatal period. Despite the decreasing rates of neonatal mortality, its importance towards decreasing the burden of under-five deaths is escalating [1]. Approximately 3 million neonates die every year due to lack of appropriate care at a global level [2] of which the highest burden (98%) occurs in the middle and low-income countries [3]. Worldwide, the key direct causes of neonatal death are preterm births (28%), neonatal infections (26%), birth asphyxia (23%) and neonatal tetanus (7%) [4]. Most of such deaths can be averted through low-cost interventions; for instance, antenatal care, appropriate care at birth and essential neonatal cares [5]. The period during labour, birth and the immediately after birth are the most critical for both maternal and newborn survival. Unfortunately, the vast majority of newborns and mothers in middle and low-income countries like Nepal do not get optimal care in this period.

As defined by World Health Organization (WHO), essential neonatal care is a comprehensive strategy developed to advance the newborn health through evidence-based interventions prior to conception, during the time of pregnancy, during delivery and soon after birth and during the postnatal period. World Health Organization (WHO) recommended essential neonatal cares are the crucial interventions to save the life of newborn. This neonatal care package includes birth preparedness and complication readiness (BPCR), four or more antenatal care (ANC) visit, skilled care at birth, social support during delivery, immediate thermal care, timely initiation of breast feeding, cord care and check-up during post partum period [6]. Newborn survival and health were not specifically addressed in the Millennium Development Goal (MDG) framework and therefore received less attention and investment. In the current era of sustainable development, social determinants are recognized as an important factor in determining the health of women and newborns. For instance, poverty, inequality, and societal unrest undermine maternal and newborn care in numerous ways and therefore identifying their association with care utilization in local context seems urgent [7].

Newborn deaths in Nepal still account for more than one-half (54%) of under-five deaths and early neonatal deaths (0–6 days) account for more than three-quarters of total neonatal deaths (79%) [8–10]. About 65% of births in rural Nepal take place at home without skilled care [11] compounded by substantial equity gaps between poor and wealthy women [12]. Newborn babies from underprivileged groups and with low socioeconomic status are dying due to the inadequate provision of neonatal care [11]. Higher rates of neonatal mortality were observed among poor socio-economic groups, Dalits and Muslims, as well as people residing in mid and far-western Nepal [13–15].

It is vital to have up-to-date evidence on the present neonatal care utilization pattern and determinants to develop a more successful program at the national and sub-national level. However, the key challenge is the lack of adequate evidence regarding essential neonatal care in specific communities like underprivileged ethnic groups residing in remote areas. Therefore, the intent of this study was to identify the patterns and determinants of neonatal care utilization among underprivileged ethnic groups in Midwest Nepal.

## Methods

### Study design and setting

This study was a mixed method in which qualitative data were embedded within the quantitative design and was conducted from September 2017 to April 2018 in Bardiya district. Bardiya district lies in Province number five, Mid Western Nepal. It is among the districts with high neonatal mortality, with a high occurrence of newborn danger signs including bacterial infections and low utilization of essential neonatal care services compared with the national status. According to the national population and housing census 2011, more than 67% of the populations in the district belong to underprivileged ethnic groups.

### Participants, sample size and sampling methods

The study followed the cluster random sampling method of women who delivered a live baby within 12 months preceding the survey. Bardiya district was divided into four clusters based on the areas defined as electoral constituency comprising five to 11 wards. Two wards from each cluster were then selected randomly. From the selected eight wards, complete enumerations of women having a child less than 1 year of age were done. Combining the record of Bacille Calmette Guerrin (BCG) immunization register, Female Community Health Volunteer (FCHV) register and discussion with FCHVs yielded a complete list of underprivileged mothers. The study considered Dalit, disadvantaged Janajati, Terai caste (excluding Terai Brahmins) and religious minorities as an underprivileged ethnic group; which was based on economic and social differences between the groups defined by Nepal government. With the estimated coverage of access to skilled care, *P* = 18% [16], 1.5- design effect, 5%- allowable error, 5% non-response rate and confidence interval of 95%, a sample size of 358 women was calculated. However, 362 women were available for interview in the sampled wards. Ten key-informant interviews were also conducted to have an in-depth understanding of essential neonatal care practices, both at the community and health facility level. For this purpose, six Auxilary Nurse Midwife were enrolled for the interview. Auxiliary Nurse Midwives were responsible for providing obstetric and newborn care at primary health care facilities, and they also know the care patterns at the community level. Further, two program focal persons (from District Public health Office); Public health Nurse and Integrated Management of Neonatal and Childhood Illness (IMNCI), were also enrolled for the interview. These focal persons were responsible for the overall management of maternal, neonatal and child health program at the facility and community level.

### Instruments and measurements

The pre-tested questionnaires were used to collect the data (see Additional file 2). Data collection tools were developed based on WHO essential neonatal care package, Demographic and Health Survey (DHS) tool and scientific articles. The tool was adapted with some modification to the national context with the help of subject experts and used in the Nepali language for actual field study. The major components of the questionnaire were essential neonatal cares, individual characteristics, household characteristics, and health service factors. Essential neonatal care utilization was the outcome variable of this study, which was taken as a composite score, created from 11 neonatal care items. Specifically, essential neonatal care includes birth preparedness and complication readiness (BPCR), four or more antenatal care (ANC) visit, skilled care at birth, social support during delivery, immediate thermal care, timely initiation of breast feeding, cord care and check-up during post partum period. The independent variables were divided into maternal, household and health facility level. Maternal characteristics includes age, education, occupation, birth order, experience of adverse pregnancy outcome and preceding birth interval. Likewise, household and health facility characteristics includes ethnicity, religion, mothers autonomy, wealth quintile, residence, travel time to nearest health facility, awareness of essential newborn care and perceived quality of maternal and neonatal services. The detail explanation of each variable is given below (See Additional file 1).

### Data management and analysis

Data entry was done in EpiData and entered data were exported to Statistical Package for Social Sciences (SPSS) version 23.0. Data cleaning was done using the technique of rearranging data in ascending and descending order. Descriptive statistics were computed to identify the maternal, household and health service related characteristics of study participants. The mean and standard deviation were calculated for continuous data like the age of mother and newborn, distance to health facility etc., while frequency and percentage and confidence interval were calculated for the categorical data. The Principal Component Analysis (PCA) was employed to generate essential neonatal care utilization status, wealth index, and women’s autonomy.

The composite index for the outcome variable was created by including the 11 neonatal care items (Table 3). The neonatal care items were then calculated as yes/no categories and later converted into dummy variables by assigning the value of “1” to “Yes” and “0” to “No”. Then Principle Component Analysis was performed after checking the co-linearity between explanatory variables, the Kaiser-Meyer-Olkin (KMO) measure of sampling adequacy and Bartlett’s Test of Sphericity. During the analysis, the relative significance of every items/variable for conducting PCA was confirmed by examining the communalities through which eight variables stayed in the PCA model, creating three principal components with eigenvalues greater than 1.0 explaining 73.27% of the entire variance. Complex structure/high loadings (> 0.4) were not found in the rotated component matrix. Inter-item consistencies between the variables were tested for the variables creating each component using the Cronbach’s alpha (all variables were > 0.7). Each principle component was sorted in the ascending order to check the presence of outliers. Finally, the continuous dependent variable (an index), was created. As the measure has a reasonably symmetrical distribution, the mean score was calculated and the pattern of neonatal care utilization was determined by dichotomizing the index based on the mean value.

Pearson’s Chi-square test (χ2) was performed to examine the association between the utilization of the essential neonatal care and the independent variables. The associated variables in Pearson’s Chi-square test (χ2) were then tested using crude odds ratio. The significant variables (*p* < 0.1) observed in the unadjusted logistic regression analysis were enrolled in final multivariate regression model to identify the adjusted odds ratio (aOR). Before being included in the multivariate analysis, the study was assessed for the collinearity of the explanatory variables by assessing the variance inflation factor score (existence of multicollinearity was considered if VIF > 10). The Hosmer and Lemeshow goodness-of-fit test was done to assess how the final regression model fit the data.

The first author carried out the key informant interviews in the Nepali language. One assistant from the study district (but not known to study participants) was recruited and oriented for note taking purpose. The first author transcribed the qualitative data on the same day of data collection and complemented with the information noted down during the interviews. Thematic content analysis [17] was used to explain the status and factors regarding neonatal care utilization. After familiarization by going through several times, transcriptions were read to understand the meaning of the content. In subsequent reviews of the transcripts, the contents of the in-depth interview were coded into categories and categories were sorted according to predefined themes of analysis. The categorized information were then summarized and presented along with the direct quotes and interpretation. Quotations illustrating the views of the key informants or in contradiction to the majority were extracted from the interview transcripts. Themes and categories of content analysis of key informant interviews were: health facility characteristics (infrastructure and logistics), neonatal care utilization status (pregnancy, delivery and postpartum period) and barriers/facilitators (socio-economic, socio-cultural, health services and others). The qualitative results obtained from thematic analysis were triangulated with the quantitative findings.

## Results

### Characteristics of the study population

Table 1 shows the maternal characteristics of 362 respondents. More than 51% of the last children were male, and more than half of the women (53%) reported being multiparous. Almost 9% of women experienced an adverse pregnancy outcome. Approximately one in two women had the preceding birth interval of less than 24 months.

**Table 1:** Maternal characteristics of the study population

Characteristics	Number (*n* = 362)	Percent
Maternal age (years)		
< 20	46	12.7
20–24	181	50.0
25–29	90	24.9
≥ 30	45	12.4
	Mean ± SD = 23.75 ± 4.306	
Education (Mother)		
No formal education	119	32.9
Primary	119	32.9
Secondary	107	29.6
Certificate and above	17	4.7
Education (Husband)		
No formal education	64	17.7
Primary	112	30.9
Secondary	124	34.3
Certificate and above	62	17.1
Occupation (Mother)		
Agriculture/homemakers	335	92.5
Labour	18	5.0
Business	6	1.7
Service	3	0.8
Occupation (Husband)		
Agriculture/homemakers	126	34.8
Labour	162	44.8
Business	53	14.6
Service	21	5.8
Birth Order		
1	170	47.0
2–3	164	45.3
3 and above	28	7.7
Experience of adverse pregnancy outcome		
No	329	90.9
Yes	33	9.1
Preceding Birth interval (*n* = 207)		
< 24	109	52.7
≥ 24	98	47.3

Table 2 presents the household and health service related characteristics of the respondents. Just over 66% of the mothers represented from the Tharu ethnic group, while the vast majority were Hindu (89%). Approximately equal proportion of study participants were found in the graded sub-categories of women’s autonomy (33% in each sub-category) and wealth quintile (20% in each sub-category). Almost 25% of mothers were from the rural residence. Just over 70% of the mothers lived within 1 hour away from health facilities with birthing services. Approximately 65% of the mothers had the perception that health facilities in their catchment area are offering good quality health services. Almost 35% of the women were aware of at least three immediate essential newborn care practices, while only 22.1% of women could name at least three newborn danger signs.

**Table 2:** Household and health services related characteristics of the study population

Characteristics	Number (*n* = 362)	Percent
Ethnicity		
Tharu	240	66.3
Dalits	75	20.7
Muslim	19	5.2
Magar	12	3.3
Others	16	4.5
Religion		
Hindu	322	89.0
Christian	21	5.8
Islam	19	5.2
Mothers autonomy		
Low	120	33.1
Medium	121	33.4
High	121	33.4
Wealth Quintile		
Poorest	64	17.7
Poorer	90	24.9
Middle	74	20.4
Richer	59	16.3
Richest	75	20.7
Residence		
Urban	265	73.2
Rural	97	26.8
Travel time to the nearest health facility (on foot)		
≤ 60 min	256	70.7
> 60 min	106	29.3
Awareness of immediate essential neonatal care and newborn danger signs		
Aware on at least three immediate essential neonatal care	128	35.4
Aware on at least three newborn danger signs	80	22.1
Perceived quality of maternal and neonatal services		
Perception of good quality services	235	64.9
Perception of poor quality services	127	35.1

### Essential neonatal care utilization pattern

Among the component of neonatal care during pregnancy, 69.1 and 43.1% visited four or more ANC and better planned for birth and its complications, respectively. Similarly, amongst the neonatal care components during the labor and delivery period, 75.1 and 98.1% of women received skilled care and social support during labor, respectively. With regards to the components immediately after birth, 78.2% received thermal care, 65.5% started timely breastfeeding, 91.2% got clean cord care, 72.7% exclusively breastfeed, 76.0% had a bath at the appropriate time (after 24 h of birth), 77.9% received postnatal care check-up within 24 h of birth and 35.1% attended at least one postnatal contacts for newborns between days 2–7 after birth. Through using all these parameters, the composite index of essential neonatal care utilization was determined. Consequently, 58.6% (95% CI: 53.3–63.7) neonates scored equal to or above the mean value categorized as receiving good essential neonatal care (Table 3).

**Table 3:** Essential neonatal care utilization pattern

Essential neonatal care	Number (*n* = 362)	Percent	95% CI
Four or more ANC visit	250	69.1	64.0–73.8
Planed for birth and its complications	156	43.1	37.9–48.4
Skilled care at birth	272	75.1	70.4–79.5
Social support during delivery	355	98.1	96.1–99.2
Immediate thermal care	283	78.2	73.6–82.3
Timely initiation of breastfeeding	237	65.5	60.3–70.4
Cord care	330	91.2	87.7–93.9
Exclusive BF (within 28 days)	263	72.7	67.7–77.2
Appropriate bathing time (after 24 h)	275	76.0	71.2–80.3
Received PNC check-up within 24 h (Immediate PNC)	282	77.9	73.3–82.1
At least one PNC after 24 h to 7 days	127	35.1	30.2–40.2
Overall neonatal care utilization	212	58.6	53.3–63.7

The qualitative results also complemented the quantitative findings. Practices like the application of mustard oil to the umbilical cord, immediate bathing of a baby (after birth) and use of pre-lacteal feeds were prevalent, particularly among minority ethnic groups such as Muslims, Dalits and Terai Madhesi. Such practices were guided by the deeprooted cultural beliefs underpinning traditional practices. According to the opinions of most of the key informants, still there were problems in protocol-based ANC attendance, skilled care, newborn feeding practices, Kangaroo Mother Care (KMC) practices, and Postnatal care (PNC) attendance. During key informant interview, IMNCI focal person expressed:

*“In recent years, we have made notable improvements in coverage of maternal and child health care services. Nevertheless, the disparity remains in a specific community, subgroups and locations within the district. To illustrate, the Muslim community of Mohamadpur, Dalit community of Thumni, Kamaiya Basthi, Babai Chepang and Bijayanagar etc. are some of the examples where improper newborn cares are being practiced”.*

Increasing and sustaining the coverage of all the neonatal care components along the continuum of care of mother and newborn baby from pregnancy to postpartum period, therefore, remain a challenge.

### Determinants of neonatal care utilization

After adjusting in the final regression model, determinants of essential neonatal care utilization were found both at maternal and household and health facility level. Mothers with two or less than two children increased the neonatal care utilization significantly (aOR 2.06, 95% CI: 1.13–3.75) than those with the more than two children. The mothers of the household from Tharu ethnic group (aOR 2.28, 95% CI 1.33–3.91) were observed to significantly increase the utilization of neonatal care as compared to those with other ethnic groups. Similarly, households with Hindu religion were found to have higher a likelihood of being associated with care utilization in comparison to households of other religions (aOR 2.37, 95% CI 1.03–5.46). Mothers with the perception of good quality maternal and neonatal services of nearest health facility (aOR 2.66, 95% CI 1.61–4.39) had higher utilization of essential neonatal care as compared to the mothers perceiving the poor quality of maternal and neonatal services. Similarly, neonatal care utilization was found to increase significantly as awareness of immediate newborn care increased (aOR 2.22, 95% CI 1.28–3.87). However, women’s autonomy, wealth quintile, women’s education, knowledge of danger signs and distance from the facility were not significantly associated with the neonatal care utilization (Table 4).

**Table 4:** Unadjusted and adjusted relationship of explanatory variables with essential neonatal care utilization

Variables	Neonatal care utilization		Unadjusted OR (95% CI)	Adjusted OR (95% CI)
	Yes (%)	No (%)		
Birth order				
1–2	179 (63.0)	105 (37.0)	2.32 (1.39–3.87)	2.06 (1.13–3.75)^a^
3 and more	33 (42.3)	45 (57.7)		Ref
Ethnicity				
Tharu	166 (69.2)	74 (30.8)	3.71 (2.35–5.86)	2.28 (1.33–3.91)^a^
Non-Tharu	46 (37.7)	76 (62.3)		Ref.
Mothers autonomy				
High	80 (66.1)	41 (33.9)	2.23 (1.33–3.75)	1.248 (0.69–2.27)
Medium	76 (62.8)	45 (37.2)	1.93 (1.15–3.23)	1.226 (0.68–2.21)
Low	56 (46.7)	64 (53.3)		Ref.
Religion				
Hindu	201 (62.4)	121 (37.6)	4.38 (2.11–9.09)	2.37 (1.03–5.46)^a^
Others	11 (27.5)	29 (72.5)		Ref.
Wealth Quintile				
Rich	49 (65.3)	26 (34.7)	2.09 (1.18–3.70)	1.04 (0.53–2.06)
Medium	90 (67.7)	43 (32.3)	2.32 (1.44–3.76)	1.411 (0.87–2.60)
Poor	73 (47.4)	81 (52.6)		Ref.
Education of mother				
Secondary & higher	86 (69.4)	38 (30.6)	2.01 (1.27–3.18)	1.16 (0.65–2.06)
Below secondary	126 (52.9)	112 (47.1)		Ref.
Education of father				
Secondary & higher	119 (64.0)	67 (36.0)	1.59 (1.04–2.42)	1.11 (0.65–1.92)
Below secondary	93 (52.8)	83 (47.2)		Ref.
Perceived quality of MNCH services				
Good quality services	155 (66.0)	80 (34.0)	2.38 (1.53–3.70)	2.66 (1.61–4.39)^a^
Poor quality services	57 (44.9)	70 (55.1)		Ref.
Awareness of immediate essential neonatal care				
≥ 3 neonatal cares	94 (73.4)	34 (26.6)	2.72 (1.70–4.34)	2.22 (1.28–3.87)^a^
< 3 neonatal cares	118 (50.4)	116 (49.6)		Ref.
Awareness of newborn danger signs				
≥ 3 danger signs	55 (68.8)	25 (31.3)	1.75 (1.03–2.97)	1.12 (0.64–2.26)
< 3 danger signs	157 (55.7)	125 (44.3)		Ref.
Distance to HF				
≤ 60 min	155 (62.0)	95 (38.0)	1.57 (1.00–2.47)	1.40 (0.89–2.54)
> 60 min	57 (50.9)	55 (49.1)		Ref.

^a^Statistically significant at 95% CI

The qualitative findings also supported the quantitative results, and the reported causes for inadequate neonatal care practices were themed as social and cultural barriers, lack of means of transportation, low awareness and poor perception towards the services of the birthing center in their catchment area.

A vast majority of the key informants shared their feelings that most of the women, particularly residing in the disadvantaged areas, are uneducated and less aware about the possible risks of newborn problems and danger signs, and the importance of appropriate neonatal care. Traditional and spiritual beliefs were prevalent, especially in the Muslim, Dalits and Terai Madhesi (underprivileged groups in Bardiya, Nepal) communities of the district. One of the key informants said: *“Some women emphasize in giving birth at home because of their cultural beliefs about birth contamination and untouchability”*. Use of such traditional practices has been a socio-cultural, spiritual and generational practice in the community. Mothers-in-law had also practiced the particular traditional care and transferred the same expectation to their daughters-in-law. Such transferred practice remained significant in maintaining cultural safety at the community. With regard to place of delivery, seven of the 10 participants reported that disadvantaged women generally preferred to give birth at their usual residence due to convenience in the delivery and postnatal care.

Following the government’s protocol of postnatal care visits to health institutions seemed challenging due to the inappropriate transportation facility, socio-cultural differences and practices. Key informants also assume that the health services delivered from the birthing centers did not fully ensure the quality of care due to the inadequate equipment, less equipped staffs and facilities. Most of the birthing center at a peripheral level were not able to follow the standards set in the government protocols and guidelines. These birthing centers only provide normal delivery services and If complications arise, then they have to refer the woman in hospitals. One key informant said: *“Some people think that when complications arise, such cases will be referred, why should we go there? It would be five to six hours quicker to reach a district hospital or Nepalgunj instead of going to health facilities”.*

Most of the rural women are labors and agriculture workers and they focus on their daily tasks for survival, and they have no time to give more attention and care to their babies. This meant that many women who were practicing traditional neonatal cares had inadequate knowledge on the dangers signs in newborn and evidence-based neonatal care practices.

## Discussion

Overall, only 58.6% of women/neonates met all the 11 indicators for essential care. This result is similar to the study conducted in Southwest Ethiopia [18] and India [19] but it is higher than the study done in Aksum Town, North Ethiopia [20] and rural Eastern Uganda [21]. Reasons for these differences may range from the socio-economic variation among the study areas and the cultural background of the study population. Nevertheless, utilization patterns of specific neonatal care were similar to the results from previous studies from rural Nepal and Karachi, Pakistan [22, 23]. Coverage of the selected neonatal care in this study are also consistent with the national figures reported in Nepal Demographic and Health Survey 2016 [24].

Households from Tharu ethnic groups in this study were more likely to utilize essential neonatal care as compared to other minority ethnic groups. A study among indigenous and ethnic minority women In Guatemala [25] also found to be less likely to have skilled attendance at delivery. Also, study among ethnic minorities in China [26] and scheduled castes/tribes in India [27] found to utilize limited neonatal cares compared with majority ethnic groups. These differences might be due to the harmful socio-cultural practices, women’s negative perception of neonatal care services available from health institutions and low level of awareness regarding neonatal care utilization. However, more qualitative and quantitative studies are needed to explore these areas.

The likelihood of utilizing essential neonatal care among women belonging to Christian and Muslim religion was low compared with the women belonging to the Hindu religion. Nevertheless, the mixed effects of religion were observed on maternal and neonatal care utilization [28]. Studies conducted in Kerala, India [27], Pakistan and Bangladesh [29, 30] observed that religion is among the important predictors of neonatal health outcomes. Another study [31] also suggests the physical differentiation of sexes, and a requirement for women to cover up their bodies and hide their form [32], might be contributing towards the low utilization of maternal and neonatal care among Muslims. Although such a lower neonatal care practice among Muslim and Christian women could have linked to their socio-cultural condition, it suggests further studies to explore the effects of religion on neonatal care utilization.

Women who were poorly aware of essential neonatal care were less likely to utilize the essential neonatal care than women with a good awareness of neonatal cares. Other studies have shown consistent results with this finding. Studies conducted in the Benishangul Gumuz region, Ethiopia [33], Bangladesh [34] and Chitwan district, Nepal [35] revealed that women’s awareness about essential neonatal care was statistically associated with the neonatal care practice. The possible explanation could be women with awareness of essential neonatal care, are willing to perform the practices and demand the services of ENC from health facilities.

This study showed the mothers with the perception of good quality services were more likely to utilize the essential neonatal cares than the mothers with the perception of poor quality services. The studies from Nicaragua and Nigeria also showed the positive association between utilization of neonatal care and the perception towards the quality of services provided by health facilities in their catchment area [36, 37]. Likewise, a study conducted in Zambia showed the effect of poor perception on the poor utilization of skilled neonatal care [38]. Systematic review evidence also showed the consistent result as the perception of health facility maternal and child health care contribute towards the disparities in the use of maternal health care services [39]. However, this finding should be interpreted with a caution that use of only a quantitative method might not be adequate to assess the perception of mothers towards the services.

Significantly higher neonatal care was received by the first and second birth order newborns compared with higher-birth-order newborns. This is also consistent with other prior studies [18, 40]. Such a result can be the families provide particular care to the first and second baby and such trend goes down with an increase in the number of live children either due to economic problem and negligence or due to greater confidence and cumulative experience. Significantly higher rates of neonatal deaths were observed with the babies born to older mothers and those with high parity [15].

In this study, the distance from a health facility was not significant in relation to the utilization of neonatal care. This study thus supports the findings of other scholars who assert that the distance to the health facility is not an important factor in health care utilization, as is widely presumed by local health workers, administrators, and policy-makers [41, 42]. However, these findings contradict the findings of studies that have been conducted in Nepal [43], Zambia [38], and Ethiopia [44], wherein the distance to a maternity service and the lack of access to the same were reported as significant with respect to the utilization of skilled care at birth. Moreover, wealth quintiles and women’s autonomy were not significantly associated with neonatal care utilization. Nevertheless, the role of both of these factors cannot be ruled out. Studies have shown that higher wealth quintiles and better autonomy of women were indeed associated with the utilization of neonatal care [24, 45]. However, the education of mothers and husbands and their awareness of danger signs were also not significantly associated in those studies, although others have established an association [18, 46–48]. This situation may also owe to the fact that these studies included all underprivileged ethnic groups who were likely to share a similar socio-economic background.

In terms of the policy- and program-oriented implications, this study summarizes the evidence on the notion that determinants of neonatal care utilization exist at the maternal, household as well as the health facility level, thereby suggesting multifaced interventions along the continuum of care.

This study also has some limitations in so far as it has only focused on underprivileged ethnic groups of the Bardiya district. Hence, there was no scope for comparison with other ethnic groups within this district. The quantitative findings relating to neonatal care were based on reports by mothers, that might have introduced some biases, and could have been under or overreported. Further, estimating neonatal care status based on the mean score (although the index has symmetrical distribution) may have its own limitation to present the status and for comparison purpose, as different study have a different way of estimating the mean. Therefore, interpreting the findings of this study needs consideration into these limitations.

## Conclusion

The coverage of essential neonatal care was low in the study area as compared to the national target for each component. Particularly, the coverage of planned for birth and its complications, adequate breastfeeding, and PNC attendance was low. Determinants of neonatal care existed at maternal, household as well as health facility level. Ethnicity, religion, perceived quality of maternal and neonatal services, birth order and awareness on essential newborn care were identified as determinants of neonatal care utilization. These results highlight the importance of disparities in health care utilization, which exist within underprivileged ethnic groups as evidenced by the difference in neonatal care utilization between Tharu and non-Tharu underprivileged ethnic groups and Hindu and other religions. Multifaceted interventions should be made along the continuum of maternal, neonatal and child health care by incorporating all the essential neonatal care components suggested by WHO.

## Additional files

Additional file 1: Operational definition of variables for the study. (XLSX 13 kb)

Additional file 2: Study Questionnaire - English language version. (PDF 247 kb)

## Data Availability

The datasets used and/or analyzed during this study are available from the corresponding author on reasonable request.

## Abbreviations

ANC: Antenatal care
ANM: Auxiliary Nurse Midwife
aOR: Adjusted Odds Ratio
BCG: Bacille Calmette Guerrin
BPCR: Birth Preparedness and Complication Readiness
CI: Confidence Interval
DHO: District Health Office
ENC: Essential Neonatal Care
FCHV: Female Community Health Volunteer
IMNCI: Integrated Management of Neonatal and Childhood Illness
IOM: Institute of Medicine
IRB: Institutional Review Board
KMC: Kangaroo Mother Care
KMO: Kaiser-Meyer-Olkin
MCH: Maternal and Child Health
MDG: Millennium Development Goal
PCA: Principle Component Analysis
PNC: Postnatal Care
VIF: Variance Inflation Factor
WHO: World Health Organization
