# Supplementary material for: Patterns and determinants of essential neonatal care utilization among underprivileged ethnic groups in Midwest Nepal: a mixed method study
Source: BMC Pregnancy Childbirth. 2019 Aug 27;19:310. doi: 10.1186/s12884-019-2465-6 (PMC6712593; doi:10.1186/s12884-019-2465-6)
Supplement: Supplementary file 2 — Study Questionnaire - English language version. (PDF 247 kb) [file 12884_2019_2465_MOESM2_ESM.pdf]

Questionnaire

Section 1: Basic Information

Cluster number: ___ ___ ___	Woman's name: ....
Name of the last child:	Sex: Age:
Name of VDC:	Ward Number:
Interviewer's name:	Date of interview: ___ ___ /___ ___ / 2074

Section 2: Individual Characteristics

QUESTIONNAIRE	RESULT	SKIP
1. How old are you?<br>*Probe:* How old were you at your last birthday?	Age (in completed years) _ __	
2. What is your educational level?<br>3. What is your husband's educational status?	Self Husband<br>Illiterate 1 1<br>Literate only 2 2<br>Primary 3 3<br>Secondary level 4 4<br>Certificate level 5 5<br>Bachelors and above 6 6	
4. What is your occupation?<br>5. What is your husband's occupation?	Self Husband<br>Agriculture 1 1<br>Business 2 2<br>Service 3 3<br>Labour 4 4<br>House work 5 5<br>Others 6 6	
**6.** Now I would like to ask you about all the pregnancies that you have had during your life. How many children do you give birth?	Sons ☐☐ Daughters ☐☐	
**7.** Have you ever given birth to a boy or girl who was born alive but later died?<br>(*If no, probe)*	Yes 1<br>No 2 →	Qn. 9

**8.** How many children died?	Sons [ ][ ] Girls [ ][ ]	
**9.** Have you ever had a pregnancy that did not end in a live birth?	Yes 1<br>No 2 →	 Qn. 11
**10.** How many pregnancies have you had that did not end in a LB?	Pregnancy losses [ ][ ]	
**11.** What is the birth interval between the current birth and the preceding birth? (if more than one child)	[ ][ ] Month	
**12.** Now I would like to ask you about your knowledge on essential neonatal care. Did you know about essential newborn care?	Yes 1<br>No 2 →	 Qn. 15
**13.** What type of care lies under essential newborn care?<br>(More than one answer is possible)	Wrap in clean and dry cloth 1<br>Breastfeed within 1 hr after birth 2<br>Bathing after 24 hour of birth 3<br>Place the baby in skin- skin contact 4<br>Delivery with the assistance of SBA 5<br>Postnatal check up of newborn baby 6<br>Other (specify) 98	
14. Mention the source of information about essential newborn care.	TV/Radio 1<br>Family/relatives 2<br>Health professional 3<br>Others (Specify) 5	
**15.** Did you heard about newborn danger signs?	Yes 1<br>No 2 →	 Sec. 2
**16.** What newborn danger signs do you know?<br>(more than one answer is possible)	Fast breathing 1<br>Severe chest indrawing 2<br>Unable to breastfeed 3<br>Fever 4<br>Hypothermia 5<br>Skin pustules 6<br>Umbilicus pus or red to skin and 7<br>Lethargic or unconscious 8	

	Others	9	
17. Mention the source of information about newborn Danger signs	TV/Radio<br>Family/relatives<br>Health professional<br>Others (Specify)	1<br>2<br>3<br>96	

Section 3: Household Characteristics

QUESTIONNAIRE	RESULT		SKIP
1. To what ethnic group does the head of this HH belong?	Ethnic group_______________ _ __<br>Other ethnic group (*specify*)	 996	
**2.** What is your religion?	Hindu<br>Buddhist<br>Islam<br>Kirat<br>Christian<br>Other (Specify)	1<br>2<br>3<br>4<br>5<br>96	
3. What is the ownership of the house that you currently reside in?	Self Owned<br>Rented/ Not owned<br>No response	1<br>2<br>3	

4	Does your household have following items? (Read out the options)	Yes	No
4.1	Electricity	1	2
4.2	Television	1	2
4.3	Mobile telephone	1	2
4.4	Refrigerator	1	2
4.5	Computer	1	2
4.6	Fan	1	2
5	Does any member of this household own the following items?	Yes	No
5.1	Bicycle/ rickshaw	1	2
5.2	Motorcycle/ Scooter	1	2
5.3	Three wheel tempo	1	2
5.4	Car or Truck or Heavy Vehicle	1	2

6	What is the main source of drinking water for members of your household?	Piped water into house	1
		Public tap water	2
		Tube well or borehole	3
		Dug well	4
		Stone tap	5
		Others (specify)	6
		No response	88
7	What type of fuel does your household mainly use for cooking?	Electricity	1
		LPG	2
		Biogas	3
		Kerosene	4
		Wood/ Timber	5
		Others (specify)	6
		No response	88
8	Do you have a separate room which is used as a kitchen?	Yes	1
		No	2

9	How do you make decision on	Independent	Jointly	Dependent
9.1	Small household expenditures (e.g. toothpaste, batteries, etc.)?	2	1	0
9.2	Large household expenditures (e.g. television, refrigerator, etc.)?	2	1	0
9.3	Expenditures on women's clothes and jewelry?	2	1	0
9.4	Woman's employment outside the home?	2	1	0
9.5	Purchase or sale of property?	2	1	0
9.6	Children's clothes and food?	2	1	0
9.7	Where to take children in the case of illness?	2	1	0
9.8	Where to take the mother in case of illness?	2	1	0
9.9	Purchase of medicine?	2	1	0
9.10	Children's education?	2	1	0
9.11	Use of contraception?	2	1	0
9.12	Visits to relatives?	2	1	0

Section 4: Essential Neonatal Care

QUESTIONNAIRE	RESULT	SKIP
Record name of last-born child from Section 1 here ______________________<br>Use this child's name in the following questions, where indicated.		
**1.** During *(Name)* your pregnancy, Did you have ANC check up in health facility?	Yes 1<br>No 2	
**2.** How many times did you receive antenatal care during this pregnancy?	Number of times [ ][ ]<br>Don't know	
**3.** During last pregnancy did you receive following advices from the FCHV/ health worker (Self care)?<br>*Ask each options separately*	Yes No<br>Proper, balanced diet 1 2<br>Avoid alcohol & smoking during pregnancy 1 2<br>Take rest & avoid heavy work 1 2<br>Health facility delivery 1 2<br>Initiate breast feeding within one hour of birth 1 2<br>Exclusive breast feeding 1 2<br>Importance of immunization for newborn baby 1 2	
**4.** During last pregnancy, were you given an injection in the arm to prevent you and the baby from getting Tetanus?	Yes 1<br>No 2<br>Don't know 3	
**5.** During last pregnancy, how many times did you get this Tetanus injection?	Times ……………..<br>Don't Know	
**6.** What kind of preparation did you make beforehand for the delivery of *(name)*?<br><br>Anything else?	Saved money 1<br>arranged for Transport 2<br>found blood donor 3<br>Contacted health worker to help with delivery 4<br>Arranged safe delivery kit 5	

	Arranged food 6<br>Arranged clothes 7<br>Other (specify) 8<br>No preparation 9	
**7.** Who assisted with the delivery of *(Name)*? Anyone else?<br>*if FCHV not mentioned probe*<br>**7.1.** If respondent says no one assisted, probe to determine whether any adults, relatives and family members were present at the delivery.	Doctor 1<br>Nurse/midwife /ANM 2<br>Health asst./AHW 3<br>FCHVs 5<br>Relatives/ friends/family member 7<br>Others (specify)<br>*No one* 9	
**8.** Where did you give birth to (*name*)? Probing	Home…………………………………......... 1<br>District hospital... 2<br>Primary health care centre .... 3<br>Health post .... 4<br>Private Medical Sector............................... 5<br>Non-Government Sector ___ 6<br>Other (*specify*) 96	If Home delivery, go to Qn. 10
**9.** Why didn't you deliver in a health facility?	Cost too much 1<br>Facility not open 2<br>Too far/ no transportation 3<br>Don't trust/ Poor quality service 4<br>No female provider at facility 5<br>Husband/family did not allow 6<br>Security concerns 7<br>Not necessary 8<br>Not customary 9<br>Child born before reaching facility 10<br>Other (specify) 11	
**10.** When *(Name)* was born, what instrument was used to cut the umbilical cord?	New/clean blade 1<br>Used blade 2<br>Knife/ scissors 3<br>Hasiya/Khukuri 4	

	Other (specify) 7<br>Don't know 8	
**11.** Was anything placed on the stump after the umbilical cord was cut?	Yes 1<br>No 2<br>Don’t know 3	
**12**. What was placed on the stump?	Oil 1 Ghee 5<br>Ash 2 CHX 6<br>Ointment/ Other (specify) 7<br>Powder 3 Don't know . . . 8<br>Animal dung 4	
**13.** Was *(Name)* dried before the placenta was delivered?	Yes 1<br>No 2	
**14.** Was *(Name)* wrapped in cloth before the placenta was delivered?	Yes 1<br>No 2<br>Don’t Know 98	
**15.** Was *(Name)* placed on your or others (father, grandmother) belly/breast before delivery of the placenta?	Yes No Don’t Know<br>Mother 1 2 98<br>Father 1 2 98<br>Others 96	
**16.** How long after delivery was *(name)* bathed for the first time?	Hours 1 __ __<br>Days 2 __ __<br>Weeks 3 __ __<br>DK/Don’t remember 998	
**17.** Was (*name*) delivered by caesarean section? That is, did they cut your belly open to take the baby out?	Yes 1 No<br>2	
**18.** When (*name*) was born, was he/she very large, larger than average, average, smaller than average, or very small?	Very large .... 1<br>Larger than average .... 2<br>Average.. 3<br>Smaller than average.................................. 4<br>Very small.. 5	

	DK.. 8	
19. Was (name) weighted at birth?	Yes .... 1<br>No .... 2<br>DK.. 8	
20. How much did (*name*) weigh?<br><br>If a card is available, record weight from card.	From card......................1 (kg) __ . __ __ __<br><br>From recall....................2 (kg) __ . __ __ __<br><br>DK.. 98	
**21.** How long after birth did you first put *(Name)* to the breast?<br><br><br><br>**21.1.** Did you feed colostrums to (Name) baby?	Immediately 1<br>Hours 2<br>Days 3<br>DK/Don't remember 998<br>Yes<br>No	
**22.** In first 28 days after delivery, was *(Name)* given anything other than breast milk?	Yes 1<br>No 2 →	Qn. 24
**23.** What was *(Name)* given to drink?<br><br>Anything else?	Milk (other than breast milk) 1<br>Plain water 2<br>Sugar or glucose water 3<br>Gripe water 4<br>Sugar-salt-water solution 5<br>Fruit juice 6<br>Infant formula 7<br>Tea/infusions/Coffee 8<br>Honey 9<br>Other (Specify) 96	
24. Check Qn8: Was the child delivered in a health facility?<br>☐ *Yes ⇨ Continue with Qn 21* ☐ *No ⇨ Go to Qn.24*		
25. You have said that you gave birth in *(facility-* Qn8*)*. How long did you stay there after delivery?	Hours __ __ Days 2 __ __<br>Weeks __ __ Don't know 998	

26. Before you left the *(facility* Qn8*)*, did anyone check on *(name)'s* health? e.g., someone examining *(name),* checking the cord, or seeing if *(name)* is alright.	Yes 1<br>No 2	
27. Did anyone check on *(name)'s* health after you left *(facility in EC13)?*	Yes 1<br>No 2	1⇨32<br>2⇨Section 4
28. Check Qn. 8: Did a health professionals or FCHVs assist delivery?<br>☐ Yes ⇨ Continue with Qn. 29 ☐ No ⇨ Go to 31		
29. After the delivery was over and before *(person or persons in Qn8)* left you, did they check on *(name)'s* health? For example examining (name), checking the cord, or seeing if *(name)* is alright.	Yes 1<br>No 2	
30. After the *(person or persons in Qn. 8)* left you, did anyone check on the health of *(name)*?	Yes 1<br>No 2	1⇨32<br>2⇨Section 4
31. After *(name)* was delivered, did anyone check on his/her health? For example, someone examining *(name),* Checking the cord, or seeing if the baby is alright.	Yes 1<br>No 2	2⇨ Section 4
32. Did such a check happen only once or more than once?	Once 1<br>More than once 2	1⇨32.1<br>2⇨32.2
32.1 How long after delivery did that check happen?	Hours 1 __ __ Days 2 __ __<br>Weeks 3 __ __<br>DK/Don't remember 998	
32.2. How long after delivery did the 1st of these checks happen?	Hours 1 __ __<br>Days 2 __ __<br>Weeks 3 __ __<br>DK/Don't remember 998	

33. Who checked on (name)'s health at that time?	Doctor 1<br>Nurse/midwife /ANM 2<br>Health asst./AHW 3<br>FCHVs 5<br>Relatives/ friends/family member 7<br>Others (specify)	
34. Where did this check take place?	Home.. 1<br>District hospital.. 2<br>Primary health care centre .... 3<br>Health post .... 4<br>Private Medical Sector............................... 5<br>Non-Government Sector ___ 6<br>Other (*specify*) 96	
30. PNC within 24 hour	Yes………… ☐ No………	
31. PNC after 24 hour to 7 days	Yes………… ☐ No………	

Section 5: Health Service Factors

QUESTIONNAIRE	RESULT	SKIP
**Problem in accessing maternal and newborn care**		
**1.** When you and your child are sick and/or want to get health service or treatment, is each of the following a big problem or not?<br>Getting permission to go?<br>Getting money needed for service?<br>The distance to the health facility?<br>Not wanting to go alone?<br>Concern about security?	BP / not a BP<br>Getting permission 1 2<br>Getting money 1 2<br>Distance 1 2<br>Going alone 1 2<br>Security 1 2<br>1 2	
**2.** What time does it take to reach nearest health institution (BY foot)?<br>**3.** What time does it take to reach nearest Hospital (By foot)?<br>(write the time in minute)	PHC ………………<br>HP ………………<br><br>District hospital………………<br>Private hospital ………………<br>Others (Specify) ………………	

**Perceived Health services Quality**					
**4.** Now I want to ask you about your perception regarding the quality of maternal and child health services providing by the nearest health facility of your area. How much do you agree or disagree with the following statements?	Degree				
	Strongly Agree	Disagree	Neutral	Agree	Strongly Disagree
4.1 Maternal and essential neonatal services are available in health facility	5	4	3	2	1
4.2 Confident that trained provider will be available	5	4	3	2	1
4.3 Complete information given about any treatments	5	4	3	2	1
4.4 Facility workers helpful	1	2	3	4	5
4.5 Given enough time to tell the medical staff everything she wanted to	1	2	3	4	5
4.6 Medical staff listened carefully to what she had to say	1	2	3	4	5
4.7 Facility workers talked politely	5	4	3	2	1
4.8 Proper privacy of service is maintained	5	4	3	2	1
4.9 Facility was adequately clean	5	4	3	2	1
4.10 Facility has toilets with good condition	5	4	3	2	1
4.11 Drinking water was easily available in the facility	5	4	3	2	1
4.12 The health care providers for women are adequate	5	4	3	2	1

KII Guideline

Part I: Characteristics of health facility

**01**	Facility number				
Date			Time	Hr.	Min
Name and post of interviewee					
Work Experience					
Trainings related to newborn					
**02**	Name of facility				
**03**	Location of facility				
**07**	Is health facility delivery provided?				
	Separate bed for Newborn Inpatient Care				

**MAIN QUESTION**	**PROBING**
How ready is the health facility to provide newborn services?	Is a person skilled in conducting deliveries present at the facility or on call at all the times, including holidays, to provide the delivery care?<br>Was the electricity available all times in last 7 days?<br>Was there an improved water source all times?<br>Is there any access to emergency transport?
Has the facility (ies) provide following newborn services during last three months of the assessment?<br>*(also see the maternity register)*	***Neonatal care options***<br>Neonatal resuscitation<br>KMC for premature or small babies and others<br>Providers at the facility routinely initiate breast feeding within first hour of birth<br>Providers at the facility routinely initiate breast feeding within twenty four hour of birth<br>Providers at facility routinely dry and wrap newborns to keep them warm<br>CHX application<br>Postnatal health checks for newborn before leaving HF<br>Postnatal health checks for newborn after leaving HF

What is the status of practicing essential neonatal care practices, especially by underprivileged ethnic groups	**Care practices**<br>BPCR<br>ANC visit<br>Skilled care at birth (SBA assistance)<br>Social support during labour<br>Immediate thermal care<br>Breast feeding initiation<br>Clean and safe cord care<br>Exclusive breast feeding<br>Newborn bathing<br>Postnatal health checks for newborn
What are the barriers seen regarding neonatal health care utilization in this community?	Socio-economic, cultural and health system barriers seen in the community<br>How such barriers prevent from practicing recommended care practices
